# Nonclinical and clinical pharmacology evidence for cardiovascular safety of saxagliptin

**DOI:** 10.1186/s12933-017-0595-6

**Published:** 2017-09-13

**Authors:** Pia S. Pollack, Kristina D. Chadwick, David M. Smith, Martin Billger, Boaz Hirshberg, Nayyar Iqbal, David W. Boulton

**Affiliations:** 1 AstraZeneca, 1 MedImmune Way, Gaithersburg, MD 20878 USA; 2 Bristol-Myers Squibb, New Brunswick, NJ USA; 3 AstraZeneca, Cambridge, UK; 4 AstraZeneca, Gothenburg, Mölndal, Sweden; 5 MedImmune, Gaithersburg, MD USA

**Keywords:** Diabetes mellitus, Pharmacology, Heart failure, Saxagliptin

## Abstract

**Background:**

In the Saxagliptin Assessment of Vascular Outcomes Recorded in Patients with Diabetes Mellitus (SAVOR) trial in patients with type 2 diabetes mellitus (T2D) at high risk of cardiovascular (CV) disease, saxagliptin did not increase the risk for major CV adverse events. However, there was an unexpected imbalance in events of hospitalization for heart failure (hHF), one of six components of the secondary CV composite endpoint, with a greater number of events observed with saxagliptin. Here, we examined findings from nonclinical safety and clinical pharmacology studies of saxagliptin with the aim of identifying any potential signals of myocardial injury.

**Methods:**

In vitro and in vivo (rat, dog, monkey) safety pharmacology and toxicology studies evaluating the potential effects of saxagliptin and its major active metabolite, 5-hydroxy saxagliptin, on the CV system are reviewed. In addition, results from saxagliptin clinical studies are discussed: one randomized, 2-period, double-blind, placebo-controlled single-ascending-dose study (up to 100 mg); one randomized, double-blind, placebo-controlled, sequential, multiple-ascending-high-dose study (up to 400 mg/day for 14 days); and one randomized, double-blind, 4-period, 4-treatment, cross-over thorough QTc study (up to 40 mg/day for 4 days) in healthy volunteers; as well as one randomized, placebo-controlled, sequential multiple-ascending-dose study in patients with T2D (up to 50 mg/day for 14 days).

**Results:**

Neither saxagliptin nor 5-hydroxy saxagliptin affected ligand binding to receptors and ion channels (e.g. potassium channels) or action potential duration in in vitro studies. In animal toxicology studies, no changes in the cardiac conduction system, blood pressure, heart rate, contractility, heart weight, or heart histopathology were observed. In healthy participants and patients with T2D, there were no findings suggestive of myocyte injury or fluid overload. Serum chemistry abnormalities indicative of cardiac injury, nonspecific muscle damage, or fluid homeostasis changes were infrequent and balanced across treatment groups. There were no QTc changes associated with saxagliptin. No treatment-emergent adverse events suggestive of heart failure or myocardial damage were reported.

**Conclusions:**

The saxagliptin nonclinical and clinical pharmacology programs did not identify evidence of myocardial injury and/or CV harm that may have predicted or may explain the unexpected imbalance in the rate of hHF observed in SAVOR.

**Electronic supplementary material:**

The online version of this article (doi:10.1186/s12933-017-0595-6) contains supplementary material, which is available to authorized users.

## Background

Patients with type 2 diabetes mellitus (T2D) are at increased risk of developing cardiovascular (CV) disease (CVD); the CV safety of antidiabetes drugs is therefore of critical importance. Saxagliptin, an orally active, highly potent, selective dipeptidyl peptidase-4 (DPP-4) inhibitor [1, 2] was first approved in the United States in 2009 as an adjunct to diet and exercise to improve glycaemic control in adults with T2D at doses of 2.5 or 5 mg once daily [3]. The safety profile of saxagliptin is well-established, with its use in patients with T2D associated with a low risk of hypoglycaemia and weight neutrality [4]. In compliance with postmarketing requirements [5], the Saxagliptin Assessment of Vascular Outcomes Recorded in Patients with Diabetes Mellitus-Thrombolysis in Myocardial Infarction 53 trial (SAVOR-TIMI 53; NCT01107886) was conducted—a large randomized, double-blind, placebo-controlled phase 4 study in 16,492 patients with T2D at high risk of CVD [6]. The primary objective of the study was to evaluate the CV safety of saxagliptin.

The SAVOR study met the objective of the 2008 US Food and Drug Administration (FDA) Guidance for Industry on treatments for diabetes [5] by demonstrating saxagliptin was non-inferior to placebo for the primary composite major adverse CV event (MACE) endpoint of CV death, non-fatal myocardial infarction, or non-fatal ischaemic stroke (primary safety objective); hazard ratio (HR) 1.00 (95% CI 0.89, 1.12). Superiority of saxagliptin versus placebo on the composite MACE endpoint was not demonstrated (p = 0.986). For the secondary composite endpoint of non-fatal myocardial infarction, non-fatal stroke, CV death, hospitalization for heart failure (hHF), hospitalization for unstable angina, or hospitalization for coronary revascularization, no statistically significant treatment differences were observed between saxagliptin and placebo [HR 1.02 (95% CI 0.94, 1.11); nominal p = 0.66 without multiplicity adjustment]. There was, however, an imbalance in the hHF component of the secondary composite endpoint, with a greater number of events observed with saxagliptin versus placebo [HR 1.27 (95% CI 1.07, 1.51); nominal p = 0.007 without multiplicity adjustment]. This represented an excess of 61 cases among patients treated with saxagliptin (n = 289/8280; 3.5%) versus placebo (n = 228/8212; 2.8%) over the median follow-up of 2.1 years.

The imbalance in events of hHF observed with saxagliptin in SAVOR was an unexpected finding because no signal for increased CV risk (including heart failure) had been previously detected for saxagliptin in nonclinical or clinical development or in postmarketing surveillance. Despite considerable evidence supporting the CV safety of saxagliptin and other DPP-4 inhibitors, the unexpected findings of SAVOR raise questions about the CV effects of saxagliptin [7]. Patients with T2D often require treatment with multiple glucose-lowering agents to achieve and maintain glycaemic control in the long term [8]. A clear understanding of the CV safety of saxagliptin is therefore important for guiding treatment decisions, particularly in patients with comorbidities or those with multiple CV risk factors. Here, we aimed to identify any evidence of a potential signal that may have predicted or may explain the unexpected imbalance in events of hHF observed with saxagliptin in SAVOR. To this end, we interrogated the available cardiovascular safety data from the nonclinical safety and clinical pharmacology development program for saxagliptin, with specific attention to potential findings that may have been indicative of a nonclinical or early clinical signal of myocardial injury.

## Methods

### Nonclinical

Numerous nonclinical studies including in vitro and in vivo safety pharmacology and toxicology studies evaluated the potential effects of saxagliptin and 5-hydroxy saxagliptin, the major active metabolite of saxagliptin (50% the potency of the parent inhibition of DPP-4) on the CV system, amongst other endpoints. In vitro assessments included high concentrations (≤30 µM, 9.5 µg/mL) of saxagliptin and 5-hydroxy saxagliptin, which are >200 times the maximum plasma concentrations for saxagliptin (0.024 μg/mL) and 5-hydroxy saxagliptin (0.047 μg/mL) observed in healthy individuals following ingestion of the highest recommended saxagliptin therapeutic dose of 5 mg [3]. Treatment durations for in vivo oral toxicology studies were up to 2 years in rodents and up to 12 months in non-rodents using high doses, representing large multiples of human exposures [up to 2200-times based on area under the plasma concentration time curve (AUC)]. Studies were conducted in compliance with International Conference on Harmonisation and US FDA guidance documents, current industry standards, animal welfare regulations, and where appropriate, Good Laboratory Practice regulations.

### Clinical

#### Single ascending dose (SAD) study in healthy adults

In a randomized, 2-period, double-blind, placebo-controlled SAD study, 72 healthy individuals were randomized to receive single oral doses of saxagliptin 1, 2.5, 5, 10, 20, 30, 50, 75 or 100 mg (n = 6 per dose) or matching placebo (n = 2 per saxagliptin dose). In period 1, which started after a ≥10-h fast, individuals received a single dose of saxagliptin or placebo. In period 2, which occurred after a 7-day washout period, individuals received a second dose of saxagliptin or placebo after consuming a high fat breakfast. Participants who were randomized to 75 or 100 mg received their second dose 30 min before consuming the high fat breakfast. Vital sign and 12-lead electrocardiogram (ECG) measurements were collected at screening, at various times throughout the study, and at discharge. Multiple blood samples for pharmacokinetic analysis (complete concentration–time profile) were collected just prior to dosing and for a 48-h period after dosing.

#### Multiple ascending dose (MAD) study in healthy adults

In a randomized, double-blind, placebo-controlled, sequential, multiple ascending high-dose study, 50 healthy individuals were randomized to receive oral saxagliptin 40, 100, 150, 200, 300 or 400 mg/day or matching placebo for 14 days. All doses were administered 1 h before consumption of a standard American Heart Association Step 1 diet (≤30% of calories from fats) or a standard liquid meal on days 8 and 13 (BoostPlus^®^; Nestlé). In total, there were 5 dose panels consisting of 2 individuals taking saxagliptin 40 mg, 6 individuals taking saxagliptin >40 mg, and 2 individuals taking placebo. If a dose was found to be well-tolerated, individuals in the succeeding group received the next higher dose of saxagliptin or matching placebo. Vital sign and 12-lead ECG measurements were collected at screening, at various times throughout the study, and at discharge. Multiple blood samples for pharmacokinetic analysis were collected on days 1–2 and 14–15.

#### Thorough QTc (tQTc) study in healthy adults

In a randomized, double-blind, 4-period, 4-treatment, cross-over tQTc study, healthy individuals were randomized to one of four treatment sequence groups. Treatments consisted of placebo, a therapeutic dose of saxagliptin (10 mg/day), or a supratherapeutic dose of saxagliptin (40 mg/day) on days 1–4, or placebo on days 1–3 and moxifloxacin 400 mg/day on day 4 (positive control for QT prolongation). Four days of saxagliptin treatment allowed for sufficient time to reach steady state. There was a minimum 4-day washout period [≥6 times the terminal half-life [t_½_] of moxifloxacin (t_½_ = 12–15 h [9])] between ECG measurements on study drug and baseline ECG measurement before the start of the next period. 12-lead ECGs were collected in triplicate at screening, at various times throughout the study, and at discharge, and the mean of these measurements at each time point was analyzed. Serial blood samples for pharmacokinetic analysis were collected on day 4 of each period.

To account for the high degree of correlation between heart rate and QT interval, various correction formulae, including Bazett’s formula (QTcB), Fridericia’s formula (QTcF) [10], QT interval corrected for heart rate (QTc) based on linear population-based analysis (QTcP), and QT interval corrected for heart rate based on log-linear population-based analysis (QTcLogP) were applied to obtain a QT interval independent of heart rate.

If the maximum difference between the mean time-matched changes from baseline QTc after treatment with a supratherapeutic dose of saxagliptin and placebo was 4 ms for at least 1 time point, then 32 participants would provide >90% power to conclude that saxagliptin had no effect on QTc interval. A total of 42 individuals were enrolled and randomized.

#### MAD study in patients with T2D

In a randomized, placebo-controlled, sequential MAD study, 40 patients with T2D were randomized to receive 2.5, 5, 15, 30, or 50 mg/day or matching placebo for 14 days. All doses were administered after consumption of a standard breakfast. In total, there were 5 dose panels consisting of 6 patients taking saxagliptin and 2 patients taking placebo. If a dose was found to be well tolerated, patients in the succeeding group received the next higher dose of saxagliptin or matching placebo. Vital sign measurements and 12-lead ECG measurements were collected at screening, at various times throughout the study, and at discharge. Serial blood samples for pharmacokinetic analysis were collected on days 1, 7, and 14.

## Results

### Nonclinical

#### In vitro studies

The potential for saxagliptin to antagonize the binding of specific radioligands to receptors, ion channels, and enzymes was evaluated in vitro. Saxagliptin had no meaningful effect (<25% inhibition at 10 μM) on any targets assessed, including potential CV targets: adrenergic α1B and muscarinic M2 receptors, L-type Ca^2+^, GABA-A [α1B2γ2], and hNAV1.5 ion-channels, and acetylcholinesterase and phosphodiesterase-3 (Table 1) [11]. Additionally, in the human ether-a-go–go-related gene (hERG) potassium channel assay, neither saxagliptin (10 and 30 μM) nor 5-hydroxy saxagliptin (≤30 μM) significantly inhibited cardiac potassium (IKr) current. IKr current was inhibited by 5.1 ± 2.8 and 11.6 ± 4.8% with 10 and 30 μM saxagliptin, respectively. Similar results were obtained for 5-hydroxy saxagliptin. IKr current was inhibited by 3.1 ± 0.0, 3.8 ± 1.4, and 7.3 ± 1.9% with 3, 10, and 30 μM 5-hydroxy saxagliptin, respectively.

**Table 1:** Potential for saxagliptin to antagonize binding of radioligands to cardiovascular-related receptors, ion channels, and enzymes

Target	L type Ca^2+^	GABA-A α1B2γ2	Cardiac hNAV1.5	Adrenergic α1β	Muscarinic M2	ACE	PDE
IC_50_ or EC_50_, μM	>25	>30	>30	>30	>30	>60	>30

*ACE* acetylcholinesterase, *GABA-A* gamma-aminobutyric acid type A, *PDE* phosphodiesterase

The potential for saxagliptin and 5-hydroxy saxagliptin to affect action potential duration was evaluated in rabbit Purkinje fibers. Neither saxagliptin nor 5-hydroxy saxagliptin at concentrations up to 30 μM significantly affected resting membrane potential, overshoot, maximum upstroke velocity (i.e., V_max_), or time to 50% (APD_50_) and 90% (APD_90_) repolarization (Table 2).

**Table 2:** Action potential parameters in rabbit Purkinje fiber

	Control	3 μM Saxagliptin	10 μM Saxagliptin	30 μM Saxagliptin
Resting Membrane Potential, mV	−84 (1)	−84 (1)	−86 (1)	−85 (2)
% Change	0 (0)	0 (1)	2 (2)	1 (3)
Overshoot, mV	31 (1)	29 (4)	32 (4)	29 (5)
% Change	0 (0)	−7 (12)	1 (11)	−7 (14)
Maximum upstroke velocity (V_max_), v/s	471 (15)	446 (15)	462 (21)	437 (13)
% Change	0 (0)	−5 (3)	−2 (3)	−7 (5)
APD_50_, ms	149 (25)	149 (24)	153 (26)	152 (21)
% Change	0 (0)	0 (0)	2 (1)	3 (4)
APD_90_, ms	232 (11)	235 (11)	237 (14)	239 (11)
% Change	0 (0)	2 (1)	2 (1)	3 (2)

Data are mean (SEM)

*APD*_*50*_ action potential duration at 50% repolarization, *APD*_*90*_ action potential duration at 90% repolarization

Taken together, saxagliptin and 5-hydroxy saxagliptin had little effect on hERG/IKr currents and on Purkinje-fiber action potentials at concentrations up to 30 µM (≤9.5 µg/mL), suggesting that it is unlikely that either saxagliptin or 5-hydroxy saxagliptin would cause hERG-/IKr-mediated electrocardiographic effects at maximal plasma concentrations associated with the highest recommended saxagliptin therapeutic dose of 5 mg [saxagliptin (0.024 μg/mL) and 5-hydroxy saxagliptin (0.047 μg/mL)] in humans.

#### In vivo studies

The potential CV effects of intravenously (IV) administered saxagliptin was assessed in single-dose studies performed in three different animal species (rats, dogs, monkeys). Saxagliptin showed no adverse effects in rat or dog at doses ≥5.9 mg/kg, IV [11]. In monkeys, decreased blood pressure (reductions of <40 mmHg) was observed with ≥3.4 mg/kg, IV, with saxagliptin maximum observed plasma concentration (C_max_) exposures 280-times the C_max_ at the 5 mg oral human dose [11]. No adverse effects were observed in monkeys with saxagliptin at 0.225 mg/kg, IV. In a single-dose oral study in conscious dogs implanted with telemetry devices, no drug–related changes in CV parameters (P width, RR, PR, QRS, and QT intervals) were observed at a saxagliptin dose of 10 mg/kg (C_max_ approximately 125-times the 5 mg human dose) [11].

The potential CV effects of orally administered saxagliptin (and its circulating major metabolite, 5-hydroxy saxagliptin) were evaluated in single- and/or repeat-dose toxicity studies in rat, dog, and monkey. No drug-related effects on ECG (including QT intervals), blood pressure, or heart rate were observed in dogs at doses up to 25 mg/kg/day for 2 weeks or up to 10 mg/kg/day for 12 months (C_max_ exposure up to approximately 750-times the 5 mg clinical dose). Similarly, no drug-related effects on ECG, blood pressure and/or heart rate were observed in monkeys at doses up to 25 mg/kg as a single dose, 30/20 mg/kg/day after 4 weeks or up to 3 mg/kg/day for 3 months (C_max_ exposure multiples up to approximately 120-times).Transient, modest (17–19%) decreases in mean systolic blood pressure (in the absence of changes in heart rate) were observed at the beginning of the 6-month repeat-dose rat study, but were absent at the end of the dosing period (C_max_ multiples up to 780-times the 5 mg human dose). This finding is considered equivocal based on the transient nature and tail-cuff data collection method, which is inherently variable, and the lack of effect on blood pressure in females despite higher saxagliptin exposures. No increase in heart weight, indicative of heart failure, was evident in rat, dog, or monkey toxicity studies lasting up to 6 months (rat), 1 year (dog), or 3 months (monkey) in duration [11]. Taken together with no observed changes in the electrical conduction system of the heart, blood pressure, heart rate, contractility, and heart weight (no increase) or heart histopathology (e.g., cellular hypertrophy, ventricular dilatation, and/or inflammation and fibrosis if there was a myocardial injury), nonclinical data suggest no evidence of cardiac insufficiency.

### Clinical pharmacology studies

#### SAD study pharmacokinetics

Plasma exposures of saxagliptin and 5-hydroxy saxagliptin generally increased proportionally with dose. The 1 mg dose of saxagliptin could not be characterized because plasma concentrations were below the limit of detection of the assay. In the fasted and fed states, the C_max_ of saxagliptin increased proportionally up to doses of 50 mg; however, in the fed state, C_max_ increased slightly more than proportionally with saxagliptin 75 and 100 mg. AUC from time 0 extrapolated to infinity (AUC_inf_) increased proportionally with dose, regardless of food. C_max_ and AUC_inf_ of 5-hydroxy saxagliptin increased proportionally up to saxagliptin doses of 50 mg but less than proportionally with saxagliptin 75 and 100 mg. The molar ratio of 5-hydroxy saxagliptin was 3- to 7-times higher than that of saxagliptin. Mean elimination half-life values in the fed or fasted state after saxagliptin 2.5–100 mg ranged from 1.2 to 3.4 h for saxagliptin and 2.8 to 6.7 h for 5-hydroxy saxagliptin. Saxagliptin was well absorbed systemically, and elimination was mainly via renal excretion, with approximately 20 and 40% of the dose excreted as saxagliptin and 5-hydroxy saxagliptin, respectively. Mean renal clearance exceeded mean GFR, therefore, active renal secretion can be inferred as contributing to the clearance of saxagliptin. In contrast, mean renal clearance values of 5-hydroxy saxagliptin did not exceed mean GFR, indicating active renal excretion does not play a role in elimination of 5-hydroxy saxagliptin.

#### MAD study pharmacokinetics

The pharmacokinetics of saxagliptin and 5-hydroxy saxagliptin following once daily doses of saxagliptin 40–400 mg for 14 days appeared linear with respect to dose. C_max_ and the AUC in one dosing interval (AUCτ) of saxagliptin increased approximately equal to the increment in dose on days 1 and 14 (Additional file 1). Both C_max_ and AUCτ for 5-hydroxy saxagliptin appeared to increase proportionally with saxagliptin doses up to 300 mg but appeared to increase less than proportionally at the 400 mg saxagliptin dose. Within each dose group, pharmacokinetic parameters on day 1 and day 14 were similar. The molar ratio of 5-hydroxy saxagliptin was 1.7- to 3-times higher than saxagliptin. Similar to the SAD study, saxagliptin was well absorbed systemically and metabolism to 5-hydroxy saxagliptin and renal excretion of the parent drug and 5-hydroxy saxagliptin were the major elimination pathways. Evidence of active renal secretion (mean renal clearance exceeding mean GFR) of saxagliptin was observed, whereas this was not the case for 5-hydroxy saxagliptin. Once daily saxagliptin at doses up to 400 mg for 14 days did not display time-dependent pharmacokinetics nor did saxagliptin or 5-hydroxy saxagliptin inhibit or induce their own metabolism.

#### tQTc study pharmacokinetics

The pharmacokinetics of saxagliptin and 5-hydroxy saxagliptin were similar to the findings in the ascending dose study in healthy volunteers, and C_max_ and AUCτ values were generally linear with respect to dose for once daily saxagliptin 10 and 40 mg over a period of 4 days (Additional file 2).

#### MAD study in patients with T2D pharmacokinetics

The pharmacokinetics of saxagliptin and 5-hydroxy saxagliptin following once daily doses of 2.5 to 50 mg for 14 days in patients with T2D (Additional file 3) were generally similar to the multiple ascending dose study in healthy volunteers, suggesting T2D itself does not markedly alter the disposition of saxagliptin or 5-hydroxy saxagliptin and that pharmacokinetic data from healthy adults can be extrapolated to patients with T2D.

#### Participant disposition and safety

Of the participants in the SAD (N = 72), MAD (N = 50), tQTc (N = 40), and MAD T2D (N = 40) studies, ≥88% completed each study (n = 70, SAD; n = 49, MAD; n = 35, tQTc; n = 40, MAD T2D). No participants discontinued because of a CV-related adverse event (AE). AEs leading to discontinuation included severe neutropenia before saxagliptin dosing [SAD; n = 1], moderate headache [SAD; n = 1], mild rash [MAD; n = 1], serious appendicitis with moderate abdominal pain, mild diarrhea, and mild pyrexia [tQTc; n = 1], and moderate urticaria [tQTc; n = 1]. The remaining discontinuations in the tQTc study were because of positive drugs of abuse screens (n = 3).

The mean age was 31 years in the SAD, MAD and tQTc studies and 54 years in the MAD T2D study. The majority of participants in the MAD, tQTc, and MAD T2D studies were white and male, whereas in the SAD study the majority of participants were black and male.

Saxagliptin was generally well tolerated in single doses up to and including 100 mg daily (SAD) and multiple doses up to and including 400 mg (MAD) in healthy volunteers or up to and including 50 mg (MAD T2D) in patients with T2D. Similarly, saxagliptin was well tolerated at doses of 10 and 40 mg/day in healthy volunteers (tQTc). There were no deaths and 1 serious AE of severe appendicitis, accompanied by moderate abdominal pain, mild diarrhea, and mild pyrexia, that was considered unrelated to study drug (tQTc; occurred after 4 doses of placebo).

#### Vital signs and physical exam

Saxagliptin had no effect on vital sign measurements, and there were no clinically relevant physical exam findings during the studies (e.g., no increase in weight suggesting fluid overload). No clinically relevant ECG abnormalities were detected in the SAD or MAD T2D studies. First-degree AV-block occurred in 2 participants in the MAD study (n = 1: placebo and n = 1: 40 mg). Both participants had normal ECGs at discharge and no further follow-up was required.

#### Examination of laboratory abnormalities suggestive of heart failure

Marked serum chemistry abnormalities indicative of cardiac injury [creatine kinase (CK) or CK-MB isoenzyme], non-specific muscle damage [aspartate aminotransferase (AST) or lactate dehydrogenase (LDH)], or possible changes in fluid homeostasis (electrolytes) were not detected or were infrequent across all 4 studies. Elevated relative CK-MB or elevated CK occurred in 1 participant in the SAD (CK-MB: n = 1: 2.5 mg, fasted), 5 participants in the MAD (CK: n = 3: placebo; n = 1: 200 mg; n = 1: 400 mg), and no participants in the tQTc study; CK and CK-MB were not assessed in the MAD T2D study. All CK-MB or elevated CK abnormalities normalized or did not require treatment, and their incidence appeared balanced across treatment groups, including placebo. No marked abnormalities in AST were detected in any of the studies. Elevated LDH concentrations occurred in 4 participants in the SAD study (n = 2: placebo, fasted; n = 1: 5 mg, fasted; n = 1: 20 mg, fasted) but not in any of the other studies. Abnormal electrolyte levels occurred in 3 participants in the SAD study (decreased inorganic phosphorous: n = 1: 30 mg; fasted and n = 1: placebo, fed; elevated serum potassium: n = 1: placebo, fasted) but not in any of the other studies.

#### tQTc findings

The QT interval correction formula that gave the lowest absolute value for the Pearson correlation between QTc and heart rate at baseline was QTcP and was used in all subsequent QTc analyses. The Pearson correlation coefficient for QTcP was −0.052 compared with 0.326, −0.166, and −0.068 for QTcB, QTcF, and QTcLogP, respectively. The ability to detect an increase in QTc interval was confirmed with moxifloxacin. The maximum placebo-adjusted, time-matched change from baseline in QTcP for moxifloxacin 400 mg was 12.52 ms and was observed at 4 h after dosing. The one-sided lower 95% confidence bound at this time of maximum effect was 7.83 ms (i.e., >0 ms); therefore, assay sensitivity was confirmed.

The maximum time-matched, placebo-adjusted mean change from baseline in heart rate for saxagliptin was 4.5 beats/min and was observed at 4 h after dosing on day 4 with the 40 mg dose. Saxagliptin was not associated with clinically meaningful QTc prolongation at a supratherapeutic dose of 40 mg. The maximum placebo-adjusted, time-matched change from baseline in QTcP for saxagliptin 40 mg was 2.35 ms, observed 24 h after dosing. The one-sided upper 95% confidence bound at this time of maximum effect was 3.58 ms, well below the 10 ms increase needed to conclude an effect. As specified a priori, results obtained with the 40 mg dose obviated the need to analyze results obtained with the 10 mg dose. Therefore, no clinically significant effect of saxagliptin on QTc was confirmed.

Saxagliptin was not associated with an increased incidence in prolongations of QTc interval or QTc change from baseline over those observed for placebo. No saxagliptin-treated individual had a QTcP interval or time-matched QTcP change from baseline outside of the normal range (QTcP >450 ms or QTcP change from baseline >30 ms). There were 2 individuals who had a QTcP change from baseline >30 ms; 1 after treatment with placebo and 1 after treatment with placebo and moxifloxacin.

**Fig. 1:**
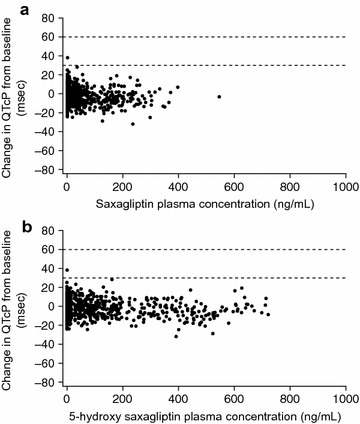
Relationship between time-matched QTcP changes from baseline and saxagliptin (**a**) and 5-hydroxy saxagliptin (**b**) plasma concentrations. Data points above the horizontal lines indicate time-matched QTcP change from baseline values >30 ms or 60 ms

Analyses of QTcP versus plasma saxagliptin and 5-hydroxy saxagliptin concentration did not show an increased incidence of QTcP intervals >450 ms or time-matched QTcP interval changes from baseline >30 ms after dosing with saxagliptin when compared with those observed after treatment with placebo (Fig. 1). Thus, no concentration-dependent effect of saxagliptin or 5-hydroxy saxagliptin on QTcP was apparent. Similarly, there was no concentration-dependent effect for either saxagliptin or 5-hydroxy saxagliptin on time-matched QTcP changes from baseline. The only time-matched QTc change from baseline >30 ms occurred in a placebo-treated individual.

#### Evaluation of AEs indicative of heart failure

CV, renal, and pulmonary system signs and symptoms that could possibly indicate myocardial damage and/or heart failure were evaluated across the SAD, MAD, MAD T2D, and tQTc studies. Treatment-emergent CV system AEs of conduction disorder (placebo, fasting), orthostatic hypotension (2.5 mg, fasting), and syncope (2.5 mg, fasting) each occurred in 1 participant in the SAD study and were not dose related. Treatment-emergent general AEs of chest pain (SAD: n = 1: 75 mg, fasted), fatigue (SAD: n = 1: 20 mg, fed; MAD: n = 2: 200 mg), and peripheral edema (MAD: n = 1: 40 mg) were infrequent and not consistent across all studies. No renal (e.g., increased serum creatinine or decreased creatinine clearance) or pulmonary system (e.g., dyspnea, orthopnea, shortness of breath) treatment-emergent AEs suggestive of heart failure or myocardial damage were reported in any of the studies.

## Discussion

In the SAVOR study, performed in patients with T2D at high risk of CVD, there was an unexpected increase in the risk for hHF among patients treated with saxagliptin versus placebo [6]. The question arises as to whether there was any signal in the saxagliptin development program that could have predicted or may explain the imbalance in hHF events seen in SAVOR. Reported here for the first time are the full results of the nonclinical and clinical pharmacology studies performed during the saxagliptin development program, which studied doses well in excess (up to 80-fold) of the highest marketed dose of saxagliptin of 5 mg. There were no findings in the nonclinical and clinical pharmacology studies suggestive of myocyte injury or fluid overload that would be predictive of an increase in clinical risk for heart failure.

In the nonclinical studies, no changes suggestive of clinically significant CV findings were observed with saxagliptin when assessed in vitro or in vivo in animals including: ligand binding to receptors and ion channels (including hERG), rabbit Purkinje fiber assessment of action potential duration, cardiovascular telemetry assessment in dog and single- and repeat-dose oral toxicology studies in multiple animal species at high doses representing large multiples of human exposures (up to 2200-times based on AUC associated with the highest human dose of 5 mg). The lack of heart weight increase, contractility change, or histopathology suggests no evidence of cardiac insufficiency in nonclinical species [11].

There is some evidence from published literature that DPP-4 inhibitors generally and saxagliptin in particular might have beneficial effects in animal models of heart failure secondary to myocardial infarction [12], isoproterenol treatment [13], pacing-induced dilated cardiomyopathy [14] and ischaemia reperfusion injury [15], as well as rodent and swine models of pressure overload secondary to transverse aortic constriction [16, 17]. The applicability of these findings to clinical heart failure remains uncertain and direct beneficial CV effects of DPP-4 inhibitors in patients with T2D are yet to be proven [7, 18]. Clinical studies of the effects of DPP-4 inhibitor treatment on vascular endothelial function, usually involving small numbers of patients, have yielded inconsistent results [19, 20] and the DPP-4 inhibitor sitagliptin did not improve echocardiographic parameters in patients with T2D and left ventricular diastolic dysfunction [21].

The CV safety profile of saxagliptin observed in nonclinical studies was corroborated by findings from early clinical pharmacology studies among participants treated with single doses of saxagliptin of up to 100 mg and doses of up to 400 mg once daily for up to 14 days. Given the absence of any suggestion of increased CV risk in the preclinical and clinical pharmacology saxagliptin program, the increase in the rate of hHF in the SAVOR study remains surprising and unexplained.

In SAVOR, the risk for hHF was increased with saxagliptin compared with placebo, but there was no increase in the overall risk for the occurrence of the primary or secondary CV composite endpoints, even among those patients with a prior history of heart failure. The degree of glycated haemoglobin (HbA1c) elevation at baseline in SAVOR was not associated with observed heart failure events [22]. There was also no clear relationship between baseline HbA1c and the risk of hHF among patients treated with saxagliptin [22]. During the course of the study, there were no significant changes in N-terminal prohormone of brain natriuretic peptide (NT-proBNP, a marker of haemodynamic stress), high-sensitivity troponin T (hs-TnT, a marker of myocardial necrosis), and high-sensitivity C-reactive protein (hs-CRP, a marker of inflammation) [23]. The absence of biomarker changes is consistent with a lack of a direct myocardial effect. Furthermore, while elevated levels of biomarkers clearly identified patients at increased CV risk, there was no biomarker that identified a particular patient population that benefited or was harmed by treatment with saxagliptin [24].

Outside of the SAVOR study, no signal for an increase in the risk of heart failure has been observed among patients treated with saxagliptin. A pooled analysis based on 20 active- and placebo-controlled Phase 2 and Phase 3 studies (N = 9156, of whom 5701 were treated with saxagliptin and 3455 were treated with placebo or active comparator) did not demonstrate an increased risk of ‘cardiac failure’ with saxagliptin [25]. The patients in these studies were at lower risk of CV events than those studied in SAVOR. There were a total of 39 heart failure events and the incidence rate ratio (saxagliptin/control) was 0.55 (95% CI 0.27, 1.12).

Observational data have also not suggested an increased risk of heart failure among patients with T2D treated with saxagliptin versus other therapies. Although observational studies have important limitations compared with randomized clinical trials such as SAVOR, including but not limited to incomplete information and potential bias [26], they do provide valuable data about drug efficacy and safety in routine clinical use. A retrospective study using a US insurance claims database (Truven Health MarketScan Commercial and Medicare Supplemental databases) found no association between hHF and treatment with a DPP-4 inhibitor relative to sulfonylureas, and no association between hHF and treatment with saxagliptin relative to sitagliptin among patients with and without baseline CVD [26]. This finding was also seen in a retrospective large cohort study conducted within the Mini-Sentinel pilot program (a program created to assist the US FDA in developing a national active safety surveillance system) where a higher risk of hHF was not observed among users of saxagliptin or sitagliptin compared with other selected glucose-lowering agents [27]. Similarly, analyses of the Taiwan National Heath Insurance database [28] and the Italian Nationwide OsMed Health-DB database [29] did not find an increased risk of hHF with saxagliptin compared to other DPP-4 inhibitors, including in patients with high CV risk.

The question arises as to whether the increased rate of hHF seen in SAVOR is a class effect or unique to saxagliptin. Most studies of DPP-4 inhibitors have shown a neutral effect on heart failure risk [30]. The findings of large CV outcome trials for other members of the DPP-4 inhibitor class, alogliptin and sitagliptin, both of which are non-DPP-4 substrate mimics, have now been reported [31, 32]. As was the case with SAVOR, these studies showed no evidence of harm for the primary MACE endpoint. With regards to hHF, the findings of these studies were inconsistent. There was an adverse trend for hHF in the Study of Examination of Cardiovascular Outcomes with Alogliptin versus Standard of Care (EXAMINE) [33], but there was no evidence of an increase in hHF risk in the Trial Evaluating Cardiovascular Outcomes with Sitagliptin (TECOS) [34]. While there are some differences in study design, including the level of CV risk in the patient population enrolled and length of study follow up, no one factor has emerged that can adequately explain the discrepant findings for hHF among the different DPP-4 inhibitor CV outcome studies. However, a reanalysis of the SAVOR, EXAMINE and TECOS trials using the difference in restricted mean survival time as a measure of CV risks, instead of the HR, found no clinically relevant differences between the three DPP-4 inhibitors and placebo in CV outcomes, including hHF [35]. Retrospective observational studies have also suggested that DPP-4 inhibitors are not associated with an increased risk of hHF compared with other antidiabetes drugs [26, 27, 29, 36] and have not identified significant intraclass differences among DPP-4 inhibitors in incidence or events of hHF [26, 28, 29].

The nonclinical and clinical pharmacology studies presented here have several limitations that should be considered when interpreting the results. Nonclinical studies were conducted in a small number of healthy animals and may not be reflective of the experience in humans with T2D. Similarly, clinical pharmacology studies involved small numbers of usually young, healthy individuals or a small number of patients with T2D (n = 40) without known CVD and were conducted over a short period of time (2 weeks or less). While the findings of these nonclinical and clinical pharmacology studies are consistent in showing no evidence of CV harm overall and specifically no evidence of a signal suggestive of heart failure risk, these studies were not designed to assess heart failure as an end point.

The data presented here, as well as results from other studies of saxagliptin specifically and DPP-4 inhibitors in general, provide reassurance of the CV safety of saxagliptin treatment in patients with T2D and the drug class overall. As many patients require more than one antiglycaemic therapy to achieve glycaemic control, the use of a combination of glucose-lowering medications with different mechanisms of action is a key therapeutic strategy as long as there are no safety implications [8]. For example, the combined use of saxagliptin with the sodium-glucose cotransporter-2 (SGLT-2) inhibitor dapagliflozin has been demonstrated to be more effective than using either agent alone in patients with T2D receiving metformin therapy who have inadequate glycaemic control, without increasing the risk of hypoglycaemia [37]. Despite the availability of multiple antidiabetes agents to help achieve and maintain glycaemic control, options are still limited among patients with diabetes who often also have renal dysfunction. In particular, the use of metformin and SGLT-2 inhibitors may not be options for patients with significant renal disease, while saxagliptin at a reduced dose of 2.5 mg can be used even in patients with severe renal impairment or end stage renal disease [3]. Saxagliptin has a well-established safety profile, with a low risk of hypoglycaemia and weight neutrality [4]. Therefore, the risks and benefits of saxagliptin should be weighed when considering saxagliptin for patients with known risk factors for heart failure [3].

## Conclusions

The results of nonclinical and clinical pharmacology studies did not identify any evidence of myocardial injury and/or CV harm associated with saxagliptin use that may have predicted or may explain the unexpected imbalance in the rate of hHF observed in SAVOR.

## Additional files

**Additional file 1.** Saxagliptin and 5-hydroxy saxagliptin pharmacokinetic parameters for multiple ascending dose study in healthy volunteers.

**Additional file 2.** Saxagliptin and 5-hydroxy saxagliptin pharmacokinetic parameters following once daily dosing for 4 days (tQTc study).

**Additional file 3.** Saxagliptin and 5-hydroxy saxagliptin pharmacokinetic parameters for multiple ascending dose study in patients with T2DM.

## Abbreviations

ACE: acetylcholinesterase
AE: adverse event
APD_50_: action potential duration at 50% repolarization
APD_90_: action potential duration at 90% repolarization
AST: aspartate aminotransferase
AUC: area under the plasma concentration–time curve
AUC_inf_: AUC from time 0 extrapolated to infinity
AUCτ: AUC in one dosing interval
AV: atrioventricular
CK: creatine kinase
C_max_: maximum observed plasma concentration
CV: cardiovascular
CVD: cardiovascular disease
DPP-4: dipeptidyl peptidase-4
ECG: electrocardiogram
eGFR: glomerular filtration rate
EXAMINE: Study of Examination of Cardiovascular Outcomes with Alogliptin versus Standard of Care
FDA: Food and Drug Administration
GABA-A: gamma-aminobutyric acid type A
HbA1c: glycated haemoglobin
hERG: human ether-a-go–go-related gene
hHF: hospitalization for heart failure
HR: hazard ratio
hs-CRP: high-sensitivity C-reactive protein
hs-TnT: high-sensitivity troponin T
IKr: cardiac potassium
IRB: Institutional Review Board
IV: intravenous
LDH: lactate dehydrogenase
MACE: major adverse CV events
MAD: multiple ascending dose
NT-proBNP: N-terminal prohormone of brain natriuretic peptide
PDE: phosphodiesterase
QTc: QT interval corrected for heart rate
QTcB: QT interval corrected with Bazett’s formula
QTcF: QT interval corrected with Fridericia’s formula
QTcLogP: QTc based on log-linear population-based analysis
QTcP: QTc based on linear population-based analysis
SAD: single ascending dose
SAVOR: Saxagliptin Assessment of Vascular Outcomes Recorded in Patients with Diabetes Mellitus
SAVOR-TIMI 53: Saxagliptin Assessment of Vascular Outcomes Recorded in Patients with Diabetes Mellitus-Thrombolysis in Myocardial Infarction 53 trial
SEM: standard error of the mean
SGLT-2: sodium-glucose cotransporter-2
TECOS: Trial Evaluating Cardiovascular Outcomes with Sitagliplin
tQTc: thorough QTc
t_½_: terminal half-life
T2D: type 2 diabetes mellitus
Vmax: maximum velocity
