# Supplementary material for: Nonclinical and clinical pharmacology evidence for cardiovascular safety of saxagliptin
Source: Cardiovasc Diabetol. 2017 Sep 13;16:113. doi: 10.1186/s12933-017-0595-6 (PMC5598064; doi:10.1186/s12933-017-0595-6)
Supplement: Supplementary file 1 — Additional file 1. Saxagliptin and 5-hydroxy saxagliptin pharmacokinetic parameters for multiple ascending dose study in healthy volunteers. [file 12933_2017_595_MOESM1_ESM.pdf]

Additional file 1. Saxagliptin and 5-hydroxy saxagliptin pharmacokinetic parameters for multiple ascending dose study in healthy volunteers

		Saxagliptin		5-Hydroxy saxagliptin	
**Pharmacokinetic parameter**	**Saxagliptin dose, mg**	**Day 1***	**Day 14†**	**Day 1***	**Day 14†**
C_{max}, ng/mL, geometric mean (CV%)	40	226 (40)	224 (33)	331 (33)	314 (40)
	100	585 (19)	487 (14)	1125 (33)	919 (14)
	150	694 (25)	614 (19)	1550 (10)	1268 (15)
	200	1207 (11)	985 (22)	1601 (24)	1389 (24)
	300	1845 (20)	1630 (31)	2622 (30)	2433 (30)
	400	2321 (18)	1863 (22)	2649 (18)	2400 (13)
AUC_{τ}, ng•h/mL, geometric mean (CV%)	40	739 (26)	800 (24)	1739 (25)	1708 (30)
	100	1899 (18)	1998 (11)	6092 (15)	5741 (14)
	150	2543 (11)	2532 (9)	7992 (13)	7474 (12)
	200	4186 (15)	4090 (10)	9479 (18)	8850 (22)
	300	6652 (22)	6439 (26)	15483 (33)	16027 (32)
	400	8364 (14)	8532 (13)	15357 (18)	16921 (12)
T_{max}, h, median (minimum, maximum)	40	1.0 (0.75, 2.00)	0.88 (0.50, 2.00)	1.50 (1.50, 3.00)	1.50 (1.50, 2.00)
	100	1.13 (0.50, 2.00)	1.50 (0.50, 2.00)	1.75 (1.00, 2.00)	1.50 (1.50, 3.00)
	150	1.50 (0.50, 2.00)	1.25 (0.75, 2.00)	2.00 (1.50, 3.00)	2.00 (1.50, 3.00)
	200	1.50 (0.50, 2.00)	1.50 (0.75, 2.00)	2.00 (1.50, 3.00)	2.00 (1.00, 3.00)
	300	1.50 (1.00, 1.50)	1.75 (1.00, 2.00)	2.00 (1.50, 3.00)	2.50 (1.50, 3.00)
	400	1.50 (1.00, 1.50)	1.50 (0.75, 2.00)	2.00 (2.00, 2.00)	2.50 (2.00, 3.00)

$T_{1/2}$, h, mean, (SD)	40	2.29 (0.18)	2.46 (0.29)	2.96 (0.29)	3.71 (1.05)
	100	2.32 (0.22)	3.03 (1.29)	4.44 (0.31)	5.86 (1.85)
	150	2.27 (0.14)	2.69 (0.91)	4.19 (0.54)	5.84 (1.96)
	200	2.25 (0.21)	3.58 (1.25)	4.08 (0.14)	5.98 (1.85)
	300	2.88 (0.85)	5.38 (3.44)	4.27 (0.32)	7.24 (2.03)
	400	3.79 (1.11)	5.48 (2.55)	4.41 (0.55)	7.38 (1.71)

AUC_{τ} area under the concentration–time curve in one dosing interval, C_{max} maximum observed plasma concentration, *CV%* coefficient of variation, *SD* standard deviation, $t_{1/2}$ half-life, T_{max} time to maximum plasma concentration. *n = 10 for saxagliptin 10 mg, n = 6 for all other doses; [†]n = 10 for saxagliptin 10 mg, n = 6 for all other doses except saxagliptin 200 mg (n = 5).
