# Supplementary material for: Nonclinical and clinical pharmacology evidence for cardiovascular safety of saxagliptin
Source: Cardiovasc Diabetol. 2017 Sep 13;16:113. doi: 10.1186/s12933-017-0595-6 (PMC5598064; doi:10.1186/s12933-017-0595-6)
Supplement: Supplementary file 2 — Additional file 2. Saxagliptin and 5-hydroxy saxagliptin pharmacokinetic parameters following once daily dosing for 4 days (tQTc study). [file 12933_2017_595_MOESM2_ESM.pdf]

Additional file 2. Saxagliptin and 5-hydroxy saxagliptin pharmacokinetic parameters following once daily dosing for 4 days (tQTc study)

Pharmacokinetic parameter	Saxagliptin dose, mg	Saxagliptin (n = 36)	5-Hydroxy saxagliptin (n = 36)
C_{max}, ng/mL, geometric mean (CV%)	10<br>40	49 (35)<br>235 (34)	118 (24)<br>497 (23)
AUC_{τ}, ng*h/mL, geometric mean (CV%)	10<br>40	171 (30)<br>745 (30)	634 (20)<br>2623 (19)
T_{max}, h, median (minimum, maximum)	10<br>40	0.75 (0.50, 3.00)<br>0.50 (0.25, 1.50)	1.50 (1.00, 3.00)<br>1.50 (1.00, 2.00)
$C_{avg[0-24]}$, ng/mL, geometric mean (CV%)	10<br>40	7 (30)<br>31 (30)	26 (20)<br>109 (19)

AUCτ area under the concentration–time curve in one dosing interval, $C_{avg[0-24]}$ average plasma concentration of saxagliptin or 5-hydroxy saxagliptin over a 24-hour dosing interval, C_{max} maximum observed plasma concentration, *CV%* coefficient of variation, T_{max} time to maximum plasma concentration.
