# Supplementary material for: Nonclinical and clinical pharmacology evidence for cardiovascular safety of saxagliptin
Source: Cardiovasc Diabetol. 2017 Sep 13;16:113. doi: 10.1186/s12933-017-0595-6 (PMC5598064; doi:10.1186/s12933-017-0595-6)
Supplement: Supplementary file 3 — Additional file 3. Saxagliptin and 5-hydroxy saxagliptin pharmacokinetic parameters for multiple ascending dose study in patients with T2DM. [file 12933_2017_595_MOESM3_ESM.pdf]

Additional file 3. Saxagliptin and 5-hydroxy saxagliptin pharmacokinetic parameters for multiple ascending dose study in patients with T2DM

		Saxagliptin		5-Hydroxy saxagliptin	
**Pharmacokinetic parameter**	**Saxagliptin dose, mg**	**Day 1***	**Day 14†**	**Day 1***	**Day 14†**
C_{max}, ng/mL, geometric mean (CV%)	2.5	11 (34)	12 (23)	35 (28)	43 (39)
	5	21 (18)	23 (22)	77 (33)	78 (31)
	15	94 (26)	89 (20)	249 (39)	243 (17)
	30	122 (33)	141 (25)	382 (32)	418 (29)
	50	206 (11)	218 (13)	1013 (36)	846 (29)
AUC_T, ng•h/mL, geometric mean (CV%)	2.5	33 (28)	34 (20)	203 (41)	251 (48)
	5	77 (25)	81 (20)	393 (44)	438 (36)
	15	371 (19)	365 (25)	1421 (43)	1395 (25)
	30	618 (40)	676 (38)	2499 (48)	2761 (51)
	50	949 (17)	915 (19)	6507 (43)	5632 (15)
T_{max}, h, median (minimum, maximum)	2.5	1.50 (0.75, 2.00)	1.50 (0.75, 2.00)	3.00 (2.00, 4.00)	3.00 (2.00, 4.00)
	5	2.00 (1.00, 3.00)	2.00 (1.50, 4.00)	3.00 (3.00, 3.00)	3.00 (2.00, 4.00)
	15	2.00 (0.75, 3.00)	1.75 (1.00, 2.00)	3.00 (2.00, 4.00)	2.50 (2.00, 3.00)
	30	3.00 (2.00, 4.00)	2.00 (1.00, 3.00)	3.50 (3.00, 4.00)	3.00 (2.00, 4.00)
	50	2.50 (1.00, 3.00)	1.50 (1.50, 3.00)	3.00 (2.00, 4.00)	3.50 (2.00, 4.00)
$T_{½}$, h, mean, (SD)	2.5	3.84 (1.72)	3.32 (1.11)	4.36 (1.17)	4.58 (1.07)
	5	2.21 (0.15)	2.33 (0.24)	3.60 (0.48)	3.67 (0.65)
	15	2.46 (0.50)	2.55 (0.35)	3.41 (0.77)	3.91 (1.90)
	30	2.35 (0.40)	2.36 (0.35)	3.53 (1.03)	3.81 (1.21)

	50	2.17 (0.27)	2.39 (0.34)	4.15 (0.97)	4.81 (0.40)

AUCτ area under the concentration–time curve in one dosing interval, C_{max} maximum observed plasma concentration, *CV%* coefficient of variation, *SD* standard deviation, $t_{½}$ half-life, T_{max} time to maximum plasma concentration. *n = 5 for saxagliptin 5 mg, n = 6 for all other doses; †n = 4 for saxagliptin 50 mg, n = 6 for all other doses.
